# The interplay between inflammation and metabolism in rheumatoid arthritis

**DOI:** 10.1038/cddis.2015.246

**Published:** 2015-09-17

**Authors:** M S Chimenti, P Triggianese, P Conigliaro, E Candi, G Melino, R Perricone

**Affiliations:** 1 Rheumatology, Allergology and Clinical Immunology, Department of “Medicina dei Sistemi”, University of Rome Tor Vergata, Rome 00133, Italy; 2 Department of Experimental Medicine and Surgery, Univeristy of Rome Tor Vergata, Rome 00133, Italy; 3 MRC Toxicology Unit, Leicester, UK

## Abstract

Rheumatoid arthritis (RA) is a chronic autoimmune disease characterized by extensive synovitis resulting in erosions of articular cartilage and marginal bone that lead to joint destruction. The autoimmune process in RA depends on the activation of immune cells, which use intracellular kinases to respond to external stimuli such as cytokines, immune complexes, and antigens. An intricate cytokine network participates in inflammation and in perpetuation of disease by positive feedback loops promoting systemic disorder. The widespread systemic effects mediated by pro-inflammatory cytokines in RA impact on metabolism and in particular in lymphocyte metabolism. Moreover, RA pathobiology seems to share some common pathways with atherosclerosis, including endothelial dysfunction that is related to underlying chronic inflammation. The extent of the metabolic changes and the types of metabolites seen may be good markers of cytokine-mediated inflammatory processes in RA. Altered metabolic fingerprints may be useful in predicting the development of RA in patients with early arthritis as well as in the evaluation of the treatment response. Evidence supports the role of metabolomic analysis as a novel and nontargeted approach for identifying potential biomarkers and for improving the clinical and therapeutical management of patients with chronic inflammatory diseases. Here, we review the metabolic changes occurring in the pathogenesis of RA as well as the implication of the metabolic features in the treatment response.

## Facts

- The elucidation of metabolic pathways in chronic inflammatory conditions, as rheumatoid arthritis (RA), give new insights on pathogenesis, clinical features and complications, and treatment outcome.
- The systemic effects mediated by pro-inflammatory cytokines in RA impact on metabolism. Moreover, RA pathobiology seems to share some common pathways with atherosclerosis including endothelial dysfunction that is related to the underlying chronic inflammation.
- In the presence of pathogens or products of inflamed tissues that provoke inflammation, macrophages and lymphocytes rapidly switch from a resting to a highly active state and exhibit a pronounced increase in production of host defence factors resulting in enhanced phagocytosis and antigen presentation.
- Highly active immune cells undergo metabolic changes with the involvement of the phosphatidylinositol- 3-OH kinase (PI(3)K)–Akt and the mechanistic target of rapamycin (mTOR) pathways.
- Differences in urine and serum metabolic profiles in patients affected by RA may be useful for the assessment of both disease activity and treatment response.

## Open Questions

- Although the autoimmune process in RA depends on the involvement of immune cells, which utilize intracellular kinases to respond to external stimuli, further research efforts are necessary in order to define more specific biomarkers to be detected in the management of that disease.
- As the metabolic profile for individual patients is highly dynamic compared with, for example, genes and protein levels, it would be worth studying how these metabolites correlate with disease activity. In the near future, the metabolite profile of individual patients can be used as an important tool for predicting the disease course and improving the treatment.
- All the oxidative damage markers correlated positively with disease activity in RA. These inflammatory pathways affect not only synovial tissues but also the endothelial structure in blood vessels leading to a vascular dysfunction. However, debated data are reported concerning the possibility of an early diagnosis of the cardiovascular (CV) involvement in RA patients. Thus, more specific biomarkers may facilitate the early detection of CV complications occurring in RA patients.
- Drugs that act on lipid/glucose metabolisms appear to confer an improvement on inflammatory features in RA patients. However, the significance of statin treatment and the effect of drugs modulating the insulin sensitivity (such as the peroxisome proliferator-activated receptor (PPAR)-*γ* agonists) in RA patients still remain unclear.

**Figure 1:**
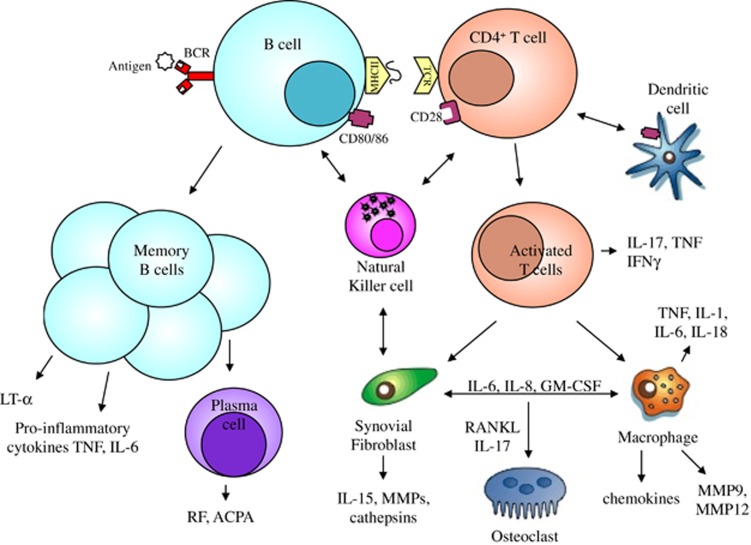
Immune pathways in rheumatoid arthritis. Innate and adaptive immune pathways integrate to promote inflammation and tissue damage. The interactions among dendritic cells, T cells and B cells occur primarily in the lymph node and generate both the autoimmune response and the activation of T cells. Upon stimulation by T cells, activated B cells differentiate into memory B cells and plasma cells producing autoantibodies such as RF and ACPA. B cells secrete pro-inflammatory cytokines and lymphotoxin (LT)-*α* that enhance inflammation and synovial lymphoneogenesis. In the synovial membrane, cell-contact interactions among T cells, natural killer cells, synovial fibroblasts, macrophages and osteoclasts generate positive feedback loops mediated by cytokines, chemokines, matrix metalloproteases (MMPs) and cathepsins that drive the chronic phase of the disease inducing tissue remodelling and damage. BCR, B cell receptor; TCR, T cell receptor; MHC, major histocompatibility complex

Rheumatoid arthritis (RA) is a chronic inflammatory and systemic disease characterized by extensive synovitis resulting in erosions of articular cartilage and marginal bone that lead to joint destruction.^1^ RA is considered an autoimmune disease since the production of the rheumatoid factor (RF), an autoantibody directed against determinants on the Fc fragment of immunoglobulin (Ig) G molecules, was first observed. The most relevant autoantibodies appear to be the anti-citrullinated protein antibodies (ACPA). Citrullination is the critical step for the recognition of several proteins (fibrin, vimentin, fibronectin, collagen type II), highly expressed in the synovial membrane during inflammation, by ACPA. The pathogenesis of RA (Figure 1) is a multistep process that starts with the development of autoimmunity, continues with local inflammation and finally induces bone destruction.^1, 2^ This stage, identified as pre-articular or lymphoid phase, can precede the clinical manifestation of the disease by as much as 10 years. Both the adaptive and the innate immune pathways are activated and contribute to the inflammatory process. An intricate cytokine network participates in the inflammation^3^ and in the perpetuation of disease by positive feedback loops promoting systemic disorders.^4^ The genetic basis of RA is extremely complex. The prevalence among siblings increases from <1%, in the general population, to 2–4%. Twin studies show a concordance rate for RA of 12–15% for monozygotic twins compared with 3.5% for dizygotic twins. Evidence of familial clustering demonstrated a prevalence from 2 to 12% in first-degree relatives of RA patients. The most important genetic risk factor for RA is found in the human leukocyte antigen (HLA) loci. In particular, the DR*β*1 chain, called 'shared epitope' (SE), is associated with the production of ACPA and with the disease.^5^ Signal transducer and activator of transcription 4 (STAT4) is a member of the STAT family of transcription factors.^6^ This molecule has a key role for the interleukin (IL)-12 signaling in T cells and natural killer (NK) cells, leading to the production of interferon (IFN)-*γ* and the differentiation of T helper (Th)1 and Th17 cells.^7^ Other candidate genes associated with RA are cytotoxic T-lymphocyte-associated antigen-4 (CTLA-4), the *α* and *β* chain of the IL-2 receptor (IL-2RA and IL-2RB), IFN regulatory factor 5 (IRF5), the locus located between tumor necrosis factor (TNF) receptor-associated factor 1 and C5 genes (TRAF1/C5), the gene near TNF-*α* -induced protein (TNFAIP3), and the co-stimulatory molecules CD40 and CD28.^7, 8^ Several environmental factors have been studied in RA and the interaction between genetic and environmental factors has been demonstrated in RA. Smoking, infections, sex hormones, birth weight, alcohol intake and socioeconomic status can modify the risk for RA.^9, 10^ The synovial membrane is a connective tissue formed by two main layers, the synovial lining and the synovial sublining. The synovial lining is composed by two types of synoviocytes, called macrophage-like and fibroblast-like synoviocytes, because of their surface marker expression and morphology. The synovial sublining is a soft, loose connective tissue that facilitates smooth movement of the joints. It contains blood and lymph vessels, nerve fibres and few cells including macrophages, fibroblasts and adipocytes.^11, 12^ In RA patients, the synovial membrane is characterized by cellular hyperplasia, increased vascularity and an infiltrate of inflammatory cells that invasively grow and destroy the adjacent cartilage and bone. The synovial hyperplasia, called 'pannus' is an increased thickening of the lining layer caused by the combination of cellular proliferation *in situ*, influx of cells from the circulation and reduced apoptosis with increased oxygen demand and consequent local hypoxia. Inadequate oxygenation drives the inflammatory response and the mechanisms of angiogenesis.^13, 14^ This process promotes further infiltration of inflammatory cells, production of inflammatory mediators and matrix degradation.^15, 16, 17^ The infiltrate in RA synovitis is composed by CD4^+^ T cells, B cells, plasma cells, NK cells, dendritic cells (DCs) and mast cells. Lymphoid aggregates of variable size and organization level are present in 50–60% of RA patients even if they are not specific of the disease. The development of the inflammatory process in RA involves many different cell types and a complex cytokine network. CD4^+^ T cells, upon activation and expansion, develop into different T helper cell subsets with different cytokine profile and distinct effector functions. Activated T cells that secrete IFN-*γ*, IL-2, IL-12, IL-18, TNF-*α* and granulocyte-macrophage colony-stimulating factor (GM-CSF), typically considered Th1 cytokines, are produced in the synovial fluid and expressed in the synovial membrane.^18^ Moreover, IL-17, which is produced by Th17 and mast cells, has been detected in RA synovial fluid.^19^ These cytokines activate macrophages to secrete other pro-inflammatory cytokines such as IL-1*β*,^20^ IL-6, TNF-*α* and IL-12, induce the nuclear factor (NF)-κB ligand (RANKL) expression on T cells, promote the differentiation of B cells and stimulate the release of matrix metalloproteases (MMPs) provoking the degradation of the cartilage and the activation of osteoclasts leading to the bone resorption.^21^ Macrophages are the most important source of those cytokines, however, many studies demonstrate that also cell contact interactions,^22, 23^ between synovial T lymphocytes and adjacent macrophages or fibroblasts, represent an alternative route to generate cytokines.^24^ Clinically, RA manifests with a symmetric polyarthritis characterized by synovitis that involves the small and large joints.^25^ Despite articular and periarticular manifestations being predominant, RA may affect many organs and tissues.^26^ Large epidemiological studies from the last several decades have confirmed that RA patients are 30–60% more likely to suffer from cardiovascular diseases (CVD) than subjects from the general population.^27, 28^ The impact of traditional risk factors on the development of CVD in RA is an area of active research. Disability, as measured by the Health Assessment Questionnaire (HAQ), is a predictor of both overall and CV mortality; thus, HAQ remission should be included among the major outcomes for defining remission in all RA cohorts.^29^ Collectively, evidence suggests that risk scores developed for the general population based on traditional CV risk factors, such as hypertension, type 2 diabetes mellitus (T2DM) or hyperlipidemia levels are unlikely to accurately estimate CV risk in RA, highlighting the need for RA-specific risk prediction tools.^30^ In this context, RA disease activity scores, inflammatory markers and extra-articular manifestations have repeatedly shown significant associations with metabolic disorders and increased CV risk.^28, 30^

## Metabolic Changes During Inflammation

**Figure 2:**
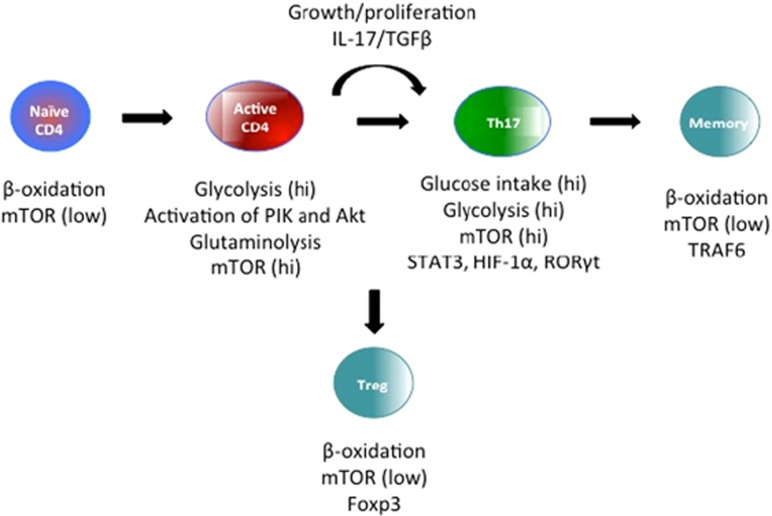
Metabolomic changes in T lymphocytes. Several metabolic changes in T cells that participate in the inflammatory process are needed to obtain polarization and activation. In T cells, metabolism is dramatically altered to support the specific needs and functions of each cell state. One of the major characteristics is the flexibility in metabolism modifications with a direct regulation of metabolic pathways by cell-extrinsic signals that drive T-cell survival, growth and proliferation. During inflammation, the function of Treg and effector T cells is subvert, resulting in the production of proinflammatory cytokines. These mechanisms have important implications for the development of cellular therapies: Treg cells can be therapeutically manipulated to enhance their function and cellular metabolism can be modified by drugs. In this context, if metabolism fails to match the demands of the cell, cell function is impaired, or cells can undergo apoptosis. Conversely, excess metabolism may prevent apoptosis, exacerbate cell function, and thus promote T-cell hyper-reactivity, and lead to autoimmunity and inflammatory diseases.^26, 29^ TGFβ: transforming growth factor-*β*; STAT3: Signal transducer and activator of transcription 3; HIF-1*α*: hypoxia-inducible factor 1*α*; ROR*γ*t: retinoic-acid-related orphan receptor-*γ*t; TRAF6: TNF receptor-associated factor 6

Metabolism is viewed simply as a mean to generate a store of energy by catabolism, and to generate macromolecules for cell maintenance and growth through anabolic pathways. The elucidation of metabolic pathways in the 20^th^ century gives insight into disorders in which there are obvious dysfunctions in metabolism, such as diabetes and atherosclerosis. Furthermore, alterations in metabolic regulation are now seen to be just as important in other diseases, such as cancer and inflammatory conditions.^31, 32, 33^ Studies on the metabolism of activated macrophages began in the 1960s. Monocytes from peritoneal exudates were shown to depend mainly on glycolysis as a source of metabolic energy, whereas *in vitro* culture of monocytes caused a significant increase in glycolysis.^34^ In 1982, a study showed that in an *in vivo* model (graft *versus* host) of immune activation, lymphocytes exhibited increased glycolysis, which was shown to be important for proliferation, together with a marked increase in glutamine use.^35^ Activated macrophages have been shown to have a high hexokinase activity, the first enzyme involved in glycolysis and in the pentose phosphate pathway. Glycolysis and glutamine metabolism are also markedly increased during phagocytosis.^36^ More recent studies have confirmed and extended these early findings. A shift towards aerobic glycolysis occurs in macrophages and DCs activated by the bacterial product lipopolysaccharide, acting through Toll-like receptor 4 (TLR4), in inflammatory macrophages and in Th17 lymphocytes.^37, 38^ On the other hand, cells that limit inflammation, such as regulatory T cells (Treg),^39^ anti-inflammatory macrophages and quiescent memory T cells that carry the CD8 antigen, exhibit oxidative metabolism with more limited rates of glycolysis.^40^ Metabolomics is the scientific study of chemical processes comprising metabolites that involves the rapid, high throughput characterization of the small molecule metabolites found in an organism. Instead of monitoring a handful of biochemicals, comprehensive biochemical profiling analyzes the change in hundreds of biochemicals including metabolites in the cells and components in the media. The metabolome is defined as those molecules with an atomic mass less than 1.5 kDa. Because no single analytical method can accommodate the chemical diversity of the entire metabolome, various methods such as nuclear magnetic resonance spectroscopy and mass spectrometry have been used, with the latter coupled to an array of separation techniques including gas and liquid chromatography.^41^ This metabolomic approach allows the analysis across different reactors over time that can help to identify and address rate-limiting media nutrients and biochemicals related to cell toxicity.^42, 43^ The feasibility of metabolomics for biomarker discovery is supported by the assumption that metabolites are important players in biological systems and that inflammation and diseases cause the disruption of biochemical pathways. Inflammation is triggered in response to tissue injury or infection, and mounting an inflammatory response is an energy-intensive process. In the presence of pathogens or the products of inflamed tissues that provoke inflammation,^44^ macrophages and lymphocytes rapidly switch from a resting state to a highly active state, exhibiting a pronounced increase in production of host defence factors, enhancing phagocytosis and antigen presentation.^45^ It is perhaps not surprising that such highly active cells undergo metabolic changes similar to those seen in tumor cells. The similarity between the metabolism that occurs in tumor cells and activated T cells has already been pointed out, with particular roles for the phosphatidylinositol- 3-OH kinase (PI(3)K)–Akt and the mechanistic target of rapamycin (mTOR) pathways, as well as the transcription factor c-myc.^46^ One difference between metabolic pathways in tumor cells and inflammatory cells is that the changes in tumor cells are driven largely by mutations, whereas those in inflammatory cells are driven by extracellular signals. In the last few years, several lines of evidence suggested that several metabolic changes in cells that participate in inflammation are needed to obtain polarization and activation of cells as lymphocytes.^47^ The products of inflamed tissues that provoke inflammation can activate T cells.^35^ Indeed, T cells are prime examples of how cell metabolism can be dramatically altered to support the specific needs and functions of each cell state. A shift towards high glycolysis is a hallmark of inflammatory cells, whereas oxidative phosphorylation is a hallmark of anti-inflammatory cells. Increased glycolysis in lymphocytes grown in culture was shown as early as 1966.^34^ This has also been shown in cells that carry the CD4 antigen, in which CD28 signalling increases glycolysis through the activation of PI(3)K and Akt.^48^ Glucose uptake and glycolysis are increased in Th17 cell *β*-oxidation (as well as Th2 and Th1 cells) compared with Treg cells, which in turn have increased membrane potential and oxidize lipids at a higher rate than other subsets of cells that carry the CD4 antigen.^49, 50^ There is, however, likely to be a gradation towards either glycolysis or oxidative metabolism because Th17 cells still exhibit some lipid oxidation. A central feature that allows this flexibility in metabolism is direct regulation of metabolic pathways by cell-extrinsic signals that drive T-cell survival, growth and proliferation.^51^ In each case, if metabolism fails to match the demands of the cell, cell function is impaired, or cell can undergo apoptosis.^52^ Conversely, excess metabolism may prevent apoptosis, exacerbate cell function, and thus promote T-cell hyper-reactivity, leading to autoimmunity and inflammatory diseases.^53, 54^ Thus, it is critical to appreciate how T-cell metabolism is regulated, and how alterations in cell metabolism impact T-cell function and fate.^55^ Metabolomic changes in T lymphocytes are summarized in Figure 2.

## Inflammation and Metabolism in RA

The widespread systemic effects mediated by pro-inflammatory cytokines in RA impact on metabolism. Many of metabolites that are particularly correlated with inflammation may contribute to the increased prevalence of metabolic syndrome (MetS) and atherosclerosis associated with RA.^56^ Thus, the extent of the metabolic changes and the types of metabolites seen may represent good markers of cytokine-mediated inflammatory process in RA. Evidences support the role of metabolomic analysis in RA patients as a novel and non-targeted approach for identifying potential biomarkers.^56, 57^ Using the metabolomic approach, the identification of several metabolites may provide insights into RA disease mechanisms and highlight their potential as markers of disease activity, metabolic and CV complications, and response to therapy.^56, 57, 58, 59^

### Metabolic syndrome and adipokines

Metabolic syndrome (MetS) is a cluster of cardiometabolic disorders that result from the increasing prevalence of obesity.^60^ The major components of MetS include insulin resistance (IR), central obesity, dyslipidemia and hypertension.^61^ It is widely accepted that MetS identifies central obesity as the main risk factor for CVD and T2DM. Various diagnostic criteria for MetS have been proposed by different organizations. Standard criteria are based on having three or more of the following five risk factors: high waist circumference (≥94 cm in men, ≥80 cm in women), high triglycerides (≥150 mg/dl), low HDL-cholesterol (<40 mg/dl in men, <50 mg/dl in women), high blood pressure (systolic ≥130 mm Hg or diastolic ≥85 mm Hg or medication use), and high blood glucose (≥100 mg/dl or presences of diabetes or medication use).^62^ Evidence concerning the prevalence of MetS in RA showed that MetS is significantly more prevalent in RA patients than in controls, and evidence documented an association between RA disease activity and MetS.^63, 64^ Thus, MetS might determine inflammatory milieu leading to the occurrence of more severe RA. Recently, it has been described that abdominal obesity is associated with high disease activity, high disability, physical inactivity and poor mental health in a cohort of 200 RA patients.^65^ Evidence clarified that adipose tissue is a dynamic endocrine organ that releases several bioactive substances including some pro-inflammatory cytokines like TNF-*α* and IL-6, and specific cytokines, termed adipokines, that may have a key role in RA pathogenesis.^28^ Adipokines were recently proposed as novel biomarkers and regulators of MetS: they are pleiotropic molecules that contribute to the so-called low-grade inflammatory state creating a cluster of metabolic aberrations. RA is associated with increased production of adipokines that are produced mainly in adipose tissue but also in synovial cells. Among the different adipokines, leptin and adiponectin were identified as relevant factors involved in interactions between metabolism and rheumatic disorders.^66^ Leptin is mainly produced by adipocytes, and its circulating levels positively correlate with white adipose tissue (WAT) mass and body mass index. Leptin has been associated with RA owing to its ability to modulate bone and cartilage metabolism although it is still unclear whether leptin can damage or protect joint structures in RA. In fact, leptin is generally considered to be pro-inflammatory; on the other hand, it has also been reported to be associated with reduced radiographic joint damage, and this effect could be related to its anabolic effects. Kontunen *et al.*^67^ registered higher leptin levels in subjects with arthritis and MetS than in patients with arthritis without MetS. This suggests that leptin is associated with MetS but not directly with arthritis, although a marked increase in plasma levels of leptin in patients with RA was noted. Moreover, in RA, leptin is able to modulate the activity of multiple immune cells.^68^ Besides leptin, adiponectin is another adipokine that seems to be involved in RA pathobiology. It shows anti-inflammatory, insulin-sensitizing and anti-atherogenic properties.^69^ Adiponectin levels have been found to be higher in RA patients than in healthy controls; moreover, synovial fluid levels of adiponectin were significantly higher in RA than in osteoarthritis patients.^70^ Evidence reported that in RA, adiponectin promotes inflammation through cytokine synthesis, attraction of inflammatory cells to the synovium and recruitment of prodestructive cells via chemokines, thus promoting matrix destruction at sites of cartilage invasion.^71^ Frommer *et al.*^71^ described that the different isoforms of adiponectin can induce gene expression and protein synthesis in human RA synovial fibroblasts, lymphocytes, endothelial cells and chondrocytes, supporting the concept of adiponectin being involved in the pathophysiologic modulation of RA effector cells. Recent findings suggest that visfatin, also called pre-B cell colony enhancing factor, may act as a regulator of the inflammation and joint destruction in animal models and resulted increased in serum and sinovial fluid.^72, 73, 74^ Serum and synovial fluid visfatin concentrations were reported to be higher in RA patients compared with healthy controls as well as its expression by rheumatoid synoviocytes at sites of attachment and invasion into both cartilage and bone.^75^ Evidences suggest that B-cell activating factor (BAFF), which has been shown to participate in B-cell survival and B- and T-cell maturation, is synthesized also by mature adipocytes and that the expression of its receptors is upregulated under pro-inflammatory conditions. Those findings suggest that BAFF may be considered as a new adipokine representing a link between obesity and inflammation.^76^ WAT also produces TNF-*α* and IL-6 that correlate with IR and MetS.^77^ Changes in lipid profiles in the blood of RA patients have been widely described, and have been suggested to be a major contributing factor to the accelerated atherosclerosis associated with RA.^78^ Serum levels of lipids and lactate are important discriminators of inflammatory burden in early arthritis and also reflect inflammatory disease activity in patients with synovitis.^56^ Madsen *et al.*^79^ compared the serum metabolic profile of patients with RA with that of healthy controls and patients with psoriatic arthritis (PsA) and found that RA or PsA patients could be distinguished with good specificity and sensitivity according to their metabolic profile. Recent evidences demonstrate that serum metabolic fingerprint of patients with active established RA differs from that of healthy controls.^56^ Authors report that 3-hydroxybutyrate results elevated in RA patients suggesting an increased level of lipolysis compared with controls.^56^ Also urinary metabolites can be analyzed as markers of cytokine-mediated inflammatory processes in RA. Urinary histamine have been suggested as a discriminator in urinary metabolites: the sources of the discriminanting histamine could be the mast cells at the synovial infiltrates and the histidine degradation related to the direct effects of TNF in accelerating muscle breakdown.^80, 81^ Differences in the metabolic profiles of baseline urine metabolites, such as histamine, glutamine, phenylacetic acid, xanthine, xanthurenic acid and creatinine, were demonstrated in patients with RA who had a good response to anti-TNF therapy compared with those who had not.^58^ Those findings suggest the relevance of the development of novel approaches for the optimization of the RA therapy.

### Atherosclerosis and endothelial dysfunction

RA pathobiology seems to share some common pathways with atherosclerosis, including endothelial dysfunction (ED) that is related to underlying chronic inflammation and presents in the early phases of the disease.^82^ Atherosclerosis is an inflammatory condition and starts as a response to injury favoring ED. The ED is defined as impaired endothelium-dependent blood-vessel dilation in response to a stimulus and is associated with increased expression of adhesion molecules, pro-inflammatory cytokines, pro-thrombotic factors, oxidative stress upregulation and abnormal vascular tone modulation. Over the last decade, a role for ED in the CV complications of inflammatory diseases has been hypothesized and several studies were performed in order to evaluate ED as a marker for risk of CV events in patients with chronic inflammatory diseases like RA.^83^ The inflammatory cascades consist in the release of pro-inflammatory cytokines, and reactive oxygen species (ROS) appear responsible for the association between RA and atherosclerosis. Inflammatory cells are able to generate oxidants, including superoxide, which are critical in non-specific host defense against pathogens such as bacteria, viruses and cancer cells. ROS and their byproducts are also implicated in arterial dysfunction via the inactivation of nitric oxide (NO), a potent vasodilator and antiaggregating molecule produced by the endothelium resulting in induction of vascular damage.^84^ All of the ROS cause an imbalance of redox state within the inflamed tissue, resulting in the activation of NF-κB and the transcription of several pro-inflammatory cytokines including TNFα, IL-1 and IL-6, which have key roles in the progression of RA and therefore are therapeutic drug targets.^85^ In RA, ROS have been attributed to directly contribute towards the destructive, proliferative synovitis.^86^ Synovial fluid and peripheral blood of RA patients display high levels of ROS and ROS-generated molecules that oxidize and degrade the major components of cartilage and bone, including collagen and hyaluronic acid.^87^ All the oxidative damage markers correlated positively with the disease activity score calculated on 28 joints.^86^ These inflammatory pathways affect the endothelial structure in blood vessels as well as synovial tissues in RA causing vascular dysfunction. Low circulating endothelial progenitor cells (EPCs) have been described in many conditions associated with increased CV risk, including RA. Recent evidence reports that circulating EPC counts in RA patients are reduced compared with non-RA controls and closely associated not only with bone erosion but also with ED assessed by brachial flow-mediated dilation (FMD).^88^ Authors reported that levels of asymmetric dimethylarginine (ADMA), an endogenous inhibitor of NO synthase (NOS), are elevated in the serum of RA patients and are related with the Homeostasis Model Assessment (HOMA) index.^89^ In this context, ADMA, by blocking NO generation, initiates and promotes processes involved in atherogenesis and may reflect an important pathway linking abnormal insulin metabolism with ED in RA.^89^ Evidence indirectly supports that remission in RA allows diminished CV morbidity. Patients with active RA, but not those in remission, had significantly increased levels of CV risk markers (circulating concentrations of N-terminal (NT)-probrain natriuretic peptide (BNP), hypertension, total cholesterol, reactive hyperaemia index, measures of arterial stiffness and intima media thickness -IMT) than the control group.^90^ As the IR is increased in RA patients, independent of obesity, recent studies have examined the association between IR and NT-proBNP in RA. The prevalence of IR was confirmed to be higher among RA patients than controls and IR results associated with higher, rather than the expected lower, concentrations of NT-proBNP. Moreover, authors hypothesized that this may be related to increased serum levels of IL-6 suggesting that IL-6 may be mechanistically involved in the relationship between IR and NT-proBNP in RA.^91^

## The Role of the Therapy: Metabolic Effects and New Potential Interventions in RA Treatment

The therapy management of RA rests primarily based on the use of disease-modifying antirheumatic drugs (DMARDs). These agents are commonly characterized by their capacity to reduce or reverse signs and symptoms, disability, impairment of quality of life, inability to work and progression of joint damage, and thus to interfere with the entire disease process.^92^ Methotrexate (MTX) is a potent antimetabolite drug that interferes with the metabolism of folic acid by the inhibition of dihydrofolate reductase and represents the first-line treatment in inflammatory arthritis.^93^ MTX, as compared with nonsteroidal anti-inflammatory drugs that merely alleviate temporarily the symptoms of joint inflammation, changes the course of the disease, retarding or even preventing the development of bone erosions. As TNF-*α* has a central role in the pathogenesis of RA, anti-TNF-*α* drugs are frequently used in forms of RA that are resistant to traditional therapeutic approaches and have acquired a prominent place in the management of rheumatologic conditions.^94, 95, 96, 97, 98, 99, 100^ Current immunosuppressive therapies act on both the adaptive and the innate immunity leading to an improvement on disease outcome.^101^ In this context, corticosteroids, MTX, sulfasalazine, leflunomide and cyclosporine A, which are used in the management of RA, exert a modulation of T-cell functions because of their effects on pro-inflammatory Th1-driven cytokines and on Th1/Th2 immune-mediated response. RA medications such as corticosteroids as well as nonsteroidal anti-inflammatory drugs and DMARDs may interfere with metabolic homeostasis conferring some CV risk.^102^ Data about the effect of synthetic DMARDs and/or biological DMARDs in this context remain controversial. An increasing number of studies are performed to explore the effect of antagonizing TNF-*α* and IL-6 on the CV outcomes in RA patients.^103, 104, 105^ Clinical trials concerning the metabolic effects of RA treatments are reported in Table 1. In addition, evidences reported that EPC counts were restored by anti-TNF-*α* therapy in RA patients, and interestingly, the restoration of EPC counts seems to be attenuated in patients with a higher bone erosion score compared with those with a lower bone erosion score, despite a similar improvement in disease activity.^106^ Evidences report that there were differences in the metabolic profiles of urine samples of patients with RA who responded to anti-TNF therapy compared with those who did not.^58^ Moreover, different anti-TNF-*α* agents seem to alter metabolites differently because of their specific mechanisms of actions. Those findings suggest that metabolomic techniques can predict outcome to anti-TNF therapy in patients with RA, providing a sensitivity and specificity for response that has potential clinical utility.^58^ Drugs that act on lipid/glucose metabolisms appear to confer an improvement on inflammatory features in RA patients (Table 2). In this context, recently, published evidence shows that in RA patients, statin treatment appears to reduce CV risk in primary prevention and that statin discontinuation is associated with an increased risk for CV events.^107^ However, the significance of statin treatment in RA patients still remains unclear as only very little evidence has been published. Clinical studies concerning the effect of drugs modulating the insulin sensitivity (such as the peroxisome proliferator-activated receptor (PPAR)-*γ* agonists) are ongoing in order to provide new potential treatment to improve both the inflammatory status and the CV outcome in RA patients (Table 2).

## Conclusions

The widespread systemic effects mediated by pro-inflammatory cytokines in RA impact on metabolism. Altered metabolic fingerprints may be useful in predicting the development of RA in patients with early arthritis as well as the response to the therapy.^58^ Both synthetic and biological DMARDs are reported to be effective in the treatment of inflammatory arthritis; however, although there is a very good response of some patients to certain therapy, there is also a complete lack of response in a large number of other patients.^108^ As the biological effects of those treatments and the mechanisms underlining the cell response are still not well understood, a more detailed understanding of the biochemical changes in the immune cells is required to elucidate toxicity pathways, the oxidative stress effects and the response mechanisms triggered by treatments. Therefore, the analysis of the potential effects of the drugs on the metabolome by analyzing the global metabolic changes associated with certain therapy can be a reliable goal. As the metabolic profile for individual patients is highly dynamic compared with, for example, genes and protein levels, it is relevant to study how these metabolites correlate with disease activity. It is possible that the metabolite profile of individual patients can be used as a tool for predicting the RA disease course and thereby facilitates the early diagnosis of the CV complications in order to improve the effectiveness of the treatment to be introduced.

## Figures and Tables

**Table 1:** Effects of treatments on metabolic and cardiovascular outcome in rheumatoid arthritis patients

**Drug**	**Conditions and purpose**	**Biological targets**	**Phase**	**Status**	**ClinicalTrials.gov identifiers**
Celecoxib in comparison with Naproxen and Ibuprofen	CV safety in patients with/at high risk for CV diseases	COX	IV	Active, not recruiting	NCT00346216
HCQ	Improvement in the insulin sensitivity	Antigen processing in APC	III	Completed	NCT01132118
Anti-TNF-*α* agents in comparison with triple therapy (SSZ+MTX+HCQ)	Improvement in the myocardial structure and function	TNF-*α* (others)	---	Recruiting participants	NCT01548768
Adalimumab	Impact on brachial ED and large artery stiffness	TNF-*α*	III	Recruiting participants	NCT01954381
Adalimumab or Infliximab or Etanercept	Development, deterioration or improvement in subclinical heart dysfunction	TNF-*α*	IV	Unknown	NCT01072058
Adalimumab or Infliximab or Etanercept or Certolizumab	Effects on blood pressure and ED	TNF-*α*	IV	Recruiting	NCT02132234
Tocilizumab	Improvement in markers of both ED and disease activity/inflammation	IL-6	IV	Unknown	NCT01752335
Tocilizumab (in comparison with MTX)	Improvement in the lipids, arterial stiffness, and markers of atherogenic risk	IL-6 (dihydrofolate reductase)	III	Completed	NCT00535782
Tocilizumab(in comparison with Etanercept)	Effects on the rate of CV ischemic events	IL-6 (TNF-*α*)	IV	Active, not recruiting	NCT01331837
Tasocitinib	Improvement in cholesterol metabolism	JAK	I	Completed	NCT01262118
Anakinra	Lowering HbA1c as well as changes in disease activity in RA patients with T2DM	IL-1	IV	Recruiting participants	NCT02236481

Abbreviations: APC, antigen-presenting cells; COX, cycloxigenase; CV, cardiovascular; ED, endothelial dysfunction; HbA1c, glycated hemoglobin; HCQ, hydroxychloroquine; IL, interleukin; JAK, Janus Kinase; MTX, methotrexate; RA, rheumatoid arthritis; SSZ, salazopyrin; T2DM, type 2 diabetes mellitus; TNF-*α*, tumor necrosis factor-*α*. Please refer studies by their ClinicalTrials.gov identifiers reported in the table

**Table 2:** Effects of drugs targeting metabolic and environmental factors on disease activity in rheumatoid arthritis patients

**Drug**	**Conditions and purpose**	**Biological targets**	**Phase**	**Status**	**ClinicalTrials.gov identifiers**
Rosuvastatin	Effects on progression of carotid IMT and arterial stiffness	HMG-CoA Reductase	II	Completed	NCT00555230
Atorvastatin	Effects on disease activity and HDL cholesterol	HMG-CoA Reductase	IV	Completed	NCT00356473
Lovastatin	Effects on disease activity and cholesterol	HMG-CoA Reductase	II	Completed	NCT00302952
Rosiglitazone	Effects on disease activity	PPAR*γ*	II	Completed	NCT00379600
Pioglitazone	Improvements in markers of both disease activity and HOMA index	PPAR*γ*	---	Completed	NCT00763139
Pioglitazone	Improvements in markers of both ED and disease activity/inflammation	PPAR*γ*	III	Completed	NCT00554853
Multidimensional intervention	Targeted, intensified, multidimensional intervention to prevent CVD in patients with early RA	Multifactorial	---	Recruiting participants	NCT02246257
Behavioral	Effects on comorbidities and disease activity	Behavioral	---	Active, not recruiting	NCT01315652
Full mouth disinfection plus short-term antiobiotic therapy	Effects on disease activity and periodontitis	Oral microbiota	---	Recruiting participants	NCT02096120
Doxycycline, Vancomycin	Effects on oral/intestinal microbiota and T cells, and on disease activity	Oral and intestinal microbiota	---	Completed	NCT01198509

Abbreviations: CVD, cardiovascular diseases; DMARD, disease-modifying anti-rheumatic drug; ED, endothelial dysfunction; HDL, high density lipoprotein; HOMA, homeostasis model assessment; IMT, intima-media thickness; PPAR, peroxisome proliferator-activated receptor; RA, rheumatoid arthritis. Please refer studies by their ClinicalTrials.gov identifiers reported in the Table

## Abbreviations

RA: rheumatoid arthritis
RF: rheumatoid factor
ACPA: anti-citrullinated protein antibodies
HLA: human leukocyte antigen
SE: shared epitope
STAT4: signal transducer and activator of transcription 4
IL: interleukin
NK: natural killer
IFN-*γ*: interferon-*γ*
Th: T helper
CTLA-4: T-lymphocyte associated antigen-4
IRF5: IFN regulatory factor 5
TNF-*α*: tumor necrosis factor-*α*
DCs: dendritic cells
MMPs: matrix metalloproteinases
CVD: cardiovascular diseases
HAQ: Health Assessment Questionnaire
T2DM: type 2 diabetes mellitus
TLR4: Toll-like receptor 4
NMR: nuclear magnetic resonance spectroscopy
MS: mass spectrometry
PI(3)K: phosphatidylinositol- 3-OH kinase
mTOR: mechanistic target of rapamycin
MetS: metabolic syndrome
IR: insulin resistence
SF36: 36-item Short Form Health Survey
WAT: white adipose tissue
PBEF: pre-B-cell colony enhancing factor
NAMPT: nicotinamide phosphoribosyltransferase
BAFF: B-cell activating factor
PsA: psoriatic arthritis
ED: endothelial dysfunction
ROS: reactive oxygen species
DAS: disease activity score
NO: nitric oxide
NF-*κ*B: nuclear factor *κ* B
EPCs: endothelial progenitor cells
FMD: flow-mediated dilation
ADMA: asymmetric dimethylarginine
NOS: NO synthase
HOMA: Homeostasis Model Assessment
BNP: natriuretic peptide
IMT: intima media thickness
DMARDs: disease-modifying antirheumatic drugs
MTX: methotrexate
PPAR: peroxisome proliferator-activated receptor.
